# Dimerisation of the *Drosophila* odorant coreceptor Orco

**DOI:** 10.3389/fncel.2014.00261

**Published:** 2014-08-28

**Authors:** Latha Mukunda, Sofia Lavista-Llanos, Bill S. Hansson, Dieter Wicher

**Affiliations:** Department Evolutionary Neuroethology, Max Planck Institute for Chemical EcologyJena, Germany

**Keywords:** odorant receptor, coreceptor, concatameric dimer, calmodulin, Orco channel

## Abstract

Odorant receptors (ORs) detect volatile molecules and transform this external information into an intracellular signal. Insect ORs are heteromers composed of two seven transmembrane proteins, an odor-specific OrX and a coreceptor (Orco) protein. These ORs form ligand gated cation channels that conduct also calcium. The sensitivity of the ORs is regulated by intracellular signaling cascades. Heterologously expressed Orco proteins form also non-selective cation channels that cannot be activated by odors but by synthetic agonists such as VUAA1. The stoichiometry of OR or Orco channels is unknown. In this study we engineered the simplest oligomeric construct, the Orco dimer (Orco di) and investigated its functional properties. Two Orco proteins were coupled via a 1-transmembrane protein to grant for proper orientation of both parts. The Orco di construct and Orco wild type (Orco wt) proteins were stably expressed in CHO (Chinese Hamster Ovary) cells. Their functional properties were investigated and compared by performing calcium imaging and patch clamp experiments. With calcium imaging experiments using allosteric agonist VUAA1 we demonstrate that the Orco di construct—similar to Orco wt—forms functional calcium conducting ion channel. This was supported by patch clamp experiments. The function of Orco di was seen to be modulated by CaM in a similar manner as the function of Orco wt. In addition, Orco di interacts with the OrX protein, Or22a. The properties of this complex are comparable to Or22a/Orco wt couples. Taken together, the properties of the Orco di construct are similar to those of channels formed by Orco wt proteins. Our results are thus compatible with the view that Orco wt channels are dimeric assemblies.

## Introduction

Olfactory receptors are transmembrane proteins that transfer information of external volatile molecules into an intracellular signal. Insect possess three classes of these receptors, odorant receptors (ORs), gustatory receptors (GRs) and variant ionotropic glutamate receptors (IRs). The insect OR gene family encodes proteins comprising seven transmembrane domains as G protein coupled receptors (GPCRs). Compared to vertebrate ORs, insect ORs are inversely inserted into the plasma membrane (Benton et al., 2006; Lundin et al., 2007; Smart et al., 2008) and are formed by heterodimeric complexes of a ligand binding olfactory receptor protein (OrX) and a highly conserved odorant co-receptor (Orco; Neuhaus et al., 2005; Benton et al., 2006). Insect ORs operate as ligand-gated ion channels (Sato et al., 2008; Wicher et al., 2008), which are tuned by intracellular signaling (Wicher et al., 2008; Kain et al., 2009; Deng et al., 2011; Getahun et al., 2013; Ignatious Raja et al., 2014).

Orco has a chaperone function as it supports the dendritic localization of OrX proteins (Larsson et al., 2004), and it contributes to the OR ion channel pore formation (Nichols et al., 2011; Pask et al., 2011; Nakagawa et al., 2012). For a recent report on Orco function see Stengl and Funk (2013). In the absence of OrX proteins, Orco forms a homomeric ion channel (Wicher et al., 2008; Jones et al., 2011). A FRET study demonstrated homodimeric and heterodimeric interactions between Orco and OrX proteins (German et al., 2013). It is, however, not excluded that dimers may dimerise to form tetramers as observed for orai1 channels (Penna et al., 2008). Whether Orco channels form dimers as the heteromeric OR channels remains elusive.

Channelrhodopsin (ChR) is another type of a seven transmembrane domain protein that acts as ion channel. Protein crystallization revealed a dimeric structure (Müller et al., 2011; Kato et al., 2012). The conductance pathway of ChR2 is located at the dimer interface with transmembrane helices 3 and 4 (Müller et al., 2011). Dimerisation has been observed for GPCR proteins, either as homomeric interaction of muscarinic acetylcholine receptors or as heteromeric coupling of GABA_B_ receptors (Wicher, 2010). Artificial homo as well as heterodimerization of GPCR proteins was performed by linking the C terminus of one protein to the N terminus of the other, spaced by a membrane spanning linker leading to functional constructs that were well expressed in heterologous cells (Terpager et al., 2009).

In the present study we ask whether a dimeric Orco construct would display channel properties and if so, whether these properties differ from those of Orco wild type (Orco wt) channels. For this purpose we engineered an Orco dimer (Orco di) and expressed it in Chinese hamster ovary (CHO) cells. By means of calcium imaging and patch clamp experiments we show that the dimeric construct displays similar properties as Orco wt channels.

## Materials and methods

### Synthetic dimer construct

The Orco dimer construct (Orco di, 3.6 kb) was generated by fusion of two *Drosophila melanogaster* Orco subunits as a single open reading frame into pcDNA3.1(-) mammalian expression vector (Invitrogen). To grant a correct orientation of the seven transmembrane domains of each of two Orco subunits, they were coupled with a 177 amino acid long 1-transmembrane protein human sodium channel, type I, beta subunit (SCN1B) (NM_001037.4). SCN1B was synthesized by Eurofins MWG Operon and cloned into Topo pcR2.1 vector (Invitrogen). The oligonucleotides used for generating Orco dimer contained XhoI/SacI restriction sites: Orco F 5′- GAT CTC GAG CTA TGA CAA CCT CGA TGC AGC C—3′ Orco R 5′-CGA GCT CTT TCT TGA GCT GCA CCA GCA CCA TAA AGT AGG T-3′ or NotI/ HindIII restriction sites: Orco F 5′- TTG CGG CCG CCT ATG ACA ACC TCG ATG CAG CCG AGC -3′ Orco R 5′- TCG AAG CTT GTT ACT TGA GCT GCA CCA GCA -3′. The PCR products were T: A cloned into Topo vector separately (Invitrogen). The two Orco units were then subcloned into pcDNA3.1(-) vector containing SCN1B. All the sequence analysis was done via double strand DNA sequencing at Eurofins MWG Operon. Sequence congruence was 100%.

### Cell culture and calcium imaging

CHO cell lines stably expressing Orco wt and Orco di were produced by Trenzyme Life Science Services (Konstanz, Germany) and grown in cytobox™ CHO select medium containing puromycin (Cytobox UG, Konstanz, Germany). The cells were grown on poly-L-lysine (0.01%, Sigma-Aldrich) coated coverslips. The culture conditions and transient transfection protocol for the coexpression of Or22a were done as described by Wicher et al. (2008). Cells for imaging were loaded with fura-2 by incubation in 1 ml CHO select medium containing 5 μM fura-2/acetomethylester (Molecular Probes, Invitrogen) for 30 min. Excitation of fura-2 at 340 and 380 nm was performed with a monochromator (Polychrome V, T.I.L.L. Photonics, Gräfelfing, Germany) coupled via an epifluorescence condenser into an Axioskop FS microscope (Carl Zeiss, Jena, Germany) with a water immersion objective (LUMPFL 40xW/IR/0.8; Olympus, Hamburg, Germany). Emitted light was separated by a 400-nm dichroic mirror and filtered with a 420-nm long-pass filter. Free intracellular Ca^2+^ concentration ([Ca^2+^]_i_) was calculated according to the equation [Ca^2+^]_i_ = *K*_eff_ (*R*−*R*_min_)/(*R*_max_−*R*).

*K*_eff_, *R*_min_ and *R*_max_ were determined as mentioned in Mukunda et al. (2014). Fluorescence images were acquired using a cooled CCD camera controlled by TILLVision 4.0 software (T.I.L.L. Photonics). The resolution was 640 × 480 pixels in a frame of 175 × 130 μm (40x/IR/0.8 objective). Image pairs were obtained by excitation for 150 ms at 340 nm and 380 nm; background fluorescence was subtracted. CHO cells were stimulated using VUAA1 and ethyl hexanoate via pipette.

### Western blot

We used ab65400 plasma membrane protein extraction kit (Abcam, Cambridge, UK) for extraction of CHO cells expressing Orco wt or Orco di or CHO–K1 (no Orco). For each sample, a cell pellet (1 g wet weight, culture density of ~8–9 × 10^6^) was collected by centrifugation. Equal loads of whole protein extracts were separated on 7.5% SDS- page gel and then electrophoretically transferred on to a PVDF membrane (Invitrogen). The membrane was then blocked in 5% non-fat dry milk, in TBS-T (20 mM Tris-HCl, 150 mM NaCl, 0.1% Tween, pH 7.6) for 1 h at room temperature. The membrane was subsequently incubated with primary polyclonal antibody 1:5000 against Orco (kindly provided by Leslie Vosshall) in 2.5% non-fat dry milk in TBS-T overnight at 4°C. The membrane was further washed with TBS-T and incubated with HRP linked secondary antibody 1:10000 for 1 h at room temperature. After washing the membrane in TBS-T the proteins were detected using ECL western blotting detection kit (Signal Fire™ Elite, Danvers, MA, USA). Densitometry of bands was performed using Image J package.^1^

### Patch-clamp electrophysiology

Current measurements and data acquisition from CHO cells were performed in whole cell configuration using EPC10 patch-clamp amplifier controlled by PatchMaster software (HEKA Elektronik, Lambrecht, Germany). Pipettes having resistances 3–4 MΩ were pulled from borosilicate capillaries (Sutter Instruments, Novato, CA, USA). The pipette solution for whole-cell recordings contained (in mM) 140 KCl, 4 NaCl, 1 CaCl_2_, 2 Mg-ATP, 0.05 Na-GTP, 5 EGTA, 10 HEPES (pH 7.3), and the bath solution contained (in mM) 140 NaCl, 5 KCl, 1 CaCl_2_, 1 MgCl_2_, 10 HEPES, 10 glucose (pH 7.4). For application of the agonist pneumatic picopump PV830 (World Precision Instruments, USA) was used and the cells were continuously perfused with bath solution in the recording/perfusion chamber (RC-27, Warner Instruments Inc., Hamden, CT, USA).

### Chemicals

VUAA1 (N-(4-ethylphenyl)-2-((4-ethyl-5-(3-pyridinyl)-4H-1,2,4-triazol-3-yl)thio)acetamide) was synthesized by the working group “Mass Spectrometry/Proteomics” of the Max-Planck Institute for chemical ecology (Jena, Germany). OLC12 was kindly provided by Charles Luetje. W-7 hydrochloride was purchased from Tocris bioscience (Wiesbaden-Nordenstadt, Germany). Ruthenium red (RR) and ethyl hexanoate (>99%) was purchased from Sigma Aldrich (Steinheim, Germany).

### Data analysis

The transmembrane domain prediction was performed by TTHMM server v.2.0 (CBS, Denmark) and TopPred 0.01- Topology prediction of membrane proteins (Mobyle@Pasteur, France). For electrophysiology the analysis software IgorPro (WaveMetrics, Lake Oswego, OR, USA) was used. Statistical analysis was performed in Prism 4 software (GraphPad Software, Inc., La Jolla, CA, USA). All data represent mean ± SEM.

## Results

**Figure 1:**
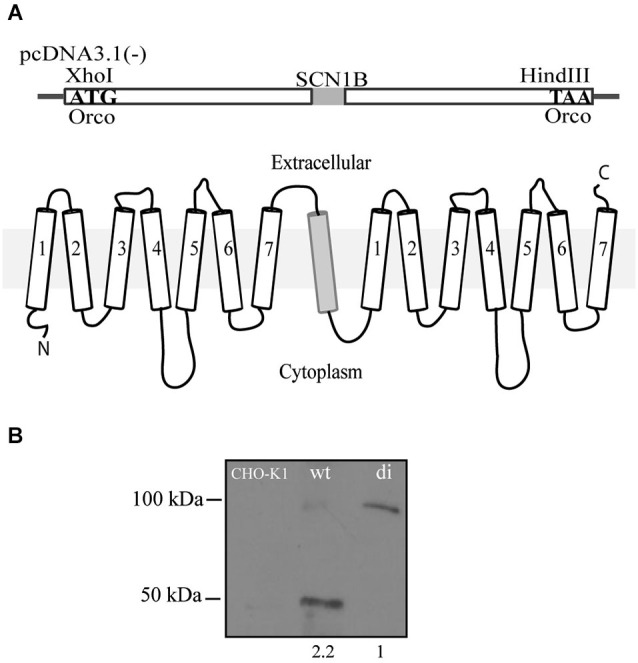
**Scheme of the Orco di construct. (A)** Two Orco subunits are fused together and spaced by the 1-transmembrane protein SCN1B to grant for a correct orientation of both Orco subunits. The 3.6 kb construct was cloned into a pcDNA3.1(-) expression vector using Xho I and Hind III restriction sites. **(B)** Western blot of CHO cells showing the expression of Orco wt and Orco di. The numbers under wt and di protein lanes indicate the relative protein levels of expression.

Orco di was generated by fusing two Orco proteins of *Drosophila melanogaster* into a single open reading frame and subsequent cloning into a pcDNA3.1(-) mammalian expression vector (Figure 1A). To grant an equal orientation of each of the seven transmembrane proteins, we coupled the single transmembrane human sodium channel beta subunit SCN1B between the two Orco subunits.

Orco wt and Orco di were stably expressed in CHO cells. To confirm that Orco wt and Orco di proteins are expressed on the membrane we extracted protein from the cells (see Section Materials and Methods) and performed a western blot. We obtained a band of the expected ~50 kDa size corresponding to Orco wt (Carraher et al., 2013) and one of the expected ~100 kDa for Orco di (Figure 1B) which notably, showed a significantly lower expression level compared to Orco wt (Figure 1B). The CHO-K1 cell membrane extract with no Orco showed no bands.

**Figure 2:**
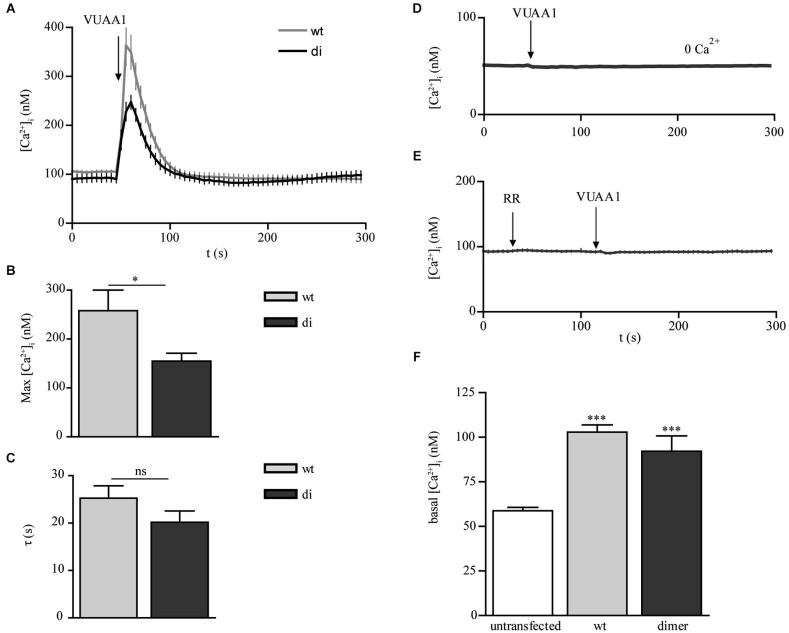
**Calcium responses to OR agonist in CHO cells expressing Orco wt and Orco di. (A)** Free intracellular calcium concentration [Ca^2+^]_i_ in Orco wt and Orco di expressing cells in response to application of VUAA1 (100 μM) (wt, *n* = 37; di, *n* = 28). **(B,C)** Comparison of maximum [Ca^2+^]_i_ **(B)** and decay time constants **(C)** in cells expressing Orco wt and Orco di as in **(A)**. **(D,E)** [Ca^2+^]_i_ in Orco di upon VUAA1 (100 μM) stimulation in **(D)** Ca^2+^ free bath solution and **(E)** in presence of Ruthenium red (0 Ca^2+^, *n* = 27; RR, 100 μM, *n* = 26). **(F)** Comparison of basal [Ca^2+^]_i_ levels in non-transfected cells (*n* = 40) and Orco wt or Orco di expressing cells as in **(A)**. Data represent mean ± SEM; unpaired *t*-test, * *p* < 0.05, *** *p* < 0.001, ns, not significant.

To study and to compare their functional properties we performed calcium imaging experiments using the ratiometric dye fura-2. Stimulation of the receptors with the synthetic Orco agonist VUAA1 (Jones et al., 2011) led to rapid increases in free intracellular Ca^2+^ concentration [Ca^2+^]_i_ for both Orco wt and di expressing cells (Figure 2A). A higher maximum increase observed in Orco wt (Figure 2B) may result from a more pronounced functional expression of the protein within the plasma membrane (see Figure 1B). For both Orco wt and Orco di the responses to VUAA1 terminated within 50 s, and the time constant *τ*, of the decay in [Ca^2+^]_i_ was not significantly different (Figure 2C). To demonstrate that the observed Ca^2+^ signals resulted from Ca^2+^ influx into the cells, we stimulated Orco di expressing cells in a Ca^2+^-free bath solution. Under these conditions [Ca^2+^]_i_ remained constant (Figure 2D). The presence of ruthenium red (RR) which has been previously shown to inhibit insect OR’s (Nakagawa et al., 2005; Sato et al., 2008; Jones et al., 2011; Nichols et al., 2011) also abolished any Ca^2+^ signal upon VUAA1 application (Figure 2E). These observations are in line with previous findings in Orco wt expressing cells (Mukunda et al., 2014) and indicate that Orco di forms RR-sensitive Ca^2+^ permeable ion channels.

Heterologously expressed Orco proteins show constitutive channel activity leading to enhanced resting [Ca^2+^]_i_ (Wicher et al., 2008). Compared with non-transfected cells the Ca^2+^ resting levels in cells expressing Orco wt and Orco di appeared to be significantly enhanced, at comparable levels (Figure 2F). Thus Orco di channels seem to show a similar constitutive activity as the Orco wt channels.

**Figure 3:**
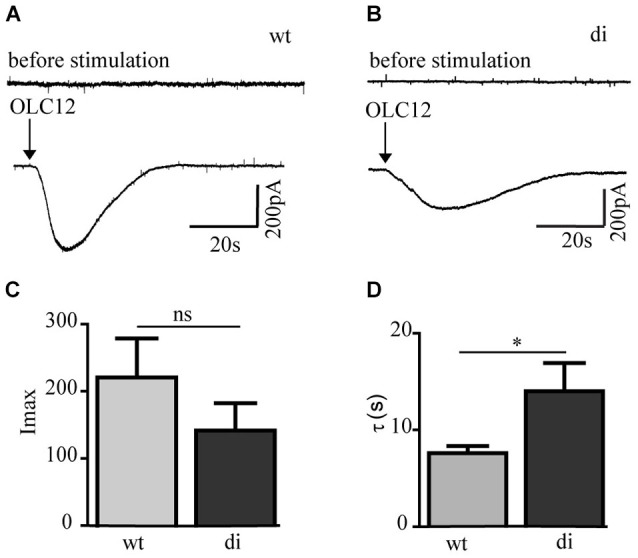
**Whole cell current responses in cells expressing Orco wt and Orco di. (A,B)** Representative current recordings in Orco wt **(A)** and Orco di **(B)** expressing cells upon stimulation with OLC12 (100 μM, arrows) at a holding potential of −60 mV. **(C,D)** Comparison of current maxima **(C)** and decay time constant **(D)** for Orco wt and Orco di expressing cells (wt, *n* = 12; di, *n* = 15). Data represent mean ± SEM; unpaired *t*-test, * *p* < 0.05, ns, not significant.

To compare the transmembrane currents conducted by Orco wt and Orco di we performed patch clamp experiments using the whole cell configuration. While receptor stimulation with VUAA1 in the non-invasive calcium imaging approach induced robust and reproducible responses, it appeared to be less efficient in the patch clamp recordings. Also for *Drosophila* Orco expression in *Xenopus* oocytes the used VUAA1 concentration of 100 μM was just above the threshold and below the EC_50_ of 190 μM (Chen and Luetje, 2012). Among the VUAA1-related OR agonists OLC12 is more potent as shown by Chen and Luetje (2012). For *Drosophila* Orco these authors report an EC_50_ of 35 μM. A comprehensive structure-activity relationship analysis of VUAA1 derivatives is presented by Taylor et al. (2012). OLC12 induced transient inward currents in Orco wt and Orco di expressing cells (Figures 3A,B). The currents induced by OLC12 had similar amplitude in Orco wt (mean 220 pA) and Orco di (mean 140 pA) expressing cells (Figure 3C). The current decay was slower for Orco di (*τ* = 14 ± 2.9 s) compared to Orco wt (*τ* = 7.6 ± 0.7 s) (Figure 3D) which indicates a slower closure of the dimer channels. In conclusion, the patch clamp measurements demonstrate that Orco di gives rise to a membrane current upon agonist stimulation.

**Figure 4:**
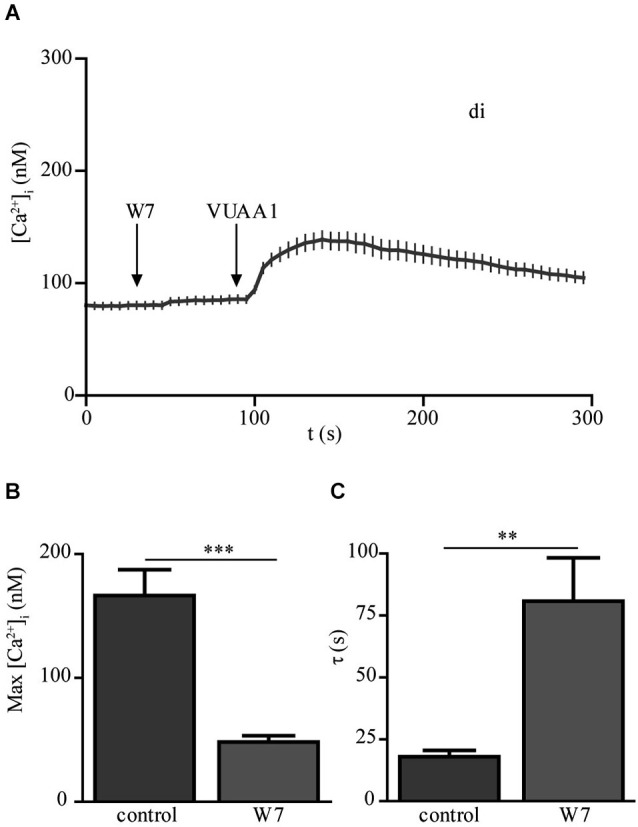
**The calmodulin inhibitor W-7 modifies the Ca^2+^ response upon OR agonist stimulation in Orco di expressing cells. (A)** [Ca^2+^]_i_ upon application of VUAA1 (100 μM) in the presence of W7 (10 μM) (*n* = 34). **(B,C)** Comparison of maximum [Ca^2+^]_i_ **(B)** and decay time constants **(C)** in cells expressing Orco di under control conditions and in presence of W7 (control, *n* = 28). Data represent mean ± SEM; unpaired *t*-test, ** *p* < 0.01, *** *p* < 0.001.

In a previous study (Mukunda et al., 2014), we have seen that calmodulin (CaM) modulates Orco channel activity. In order to check if this regulation is conserved in Orco di we stimulated cells expressing Orco di with VUAA1 in the presence of CaM inhibitor W7 (Figure 4A). Application of W7 reduced the calcium responses to VUAA1 stimulation (Figure 4B) and significantly increased the decay time constant of the Ca^2+^ response (Figure 4C). These effects are in line with the results obtained for Orco wt (Mukunda et al., 2014), and demonstrate conservation of the CaM regulation in the dimeric Orco construct.

**Figure 5:**
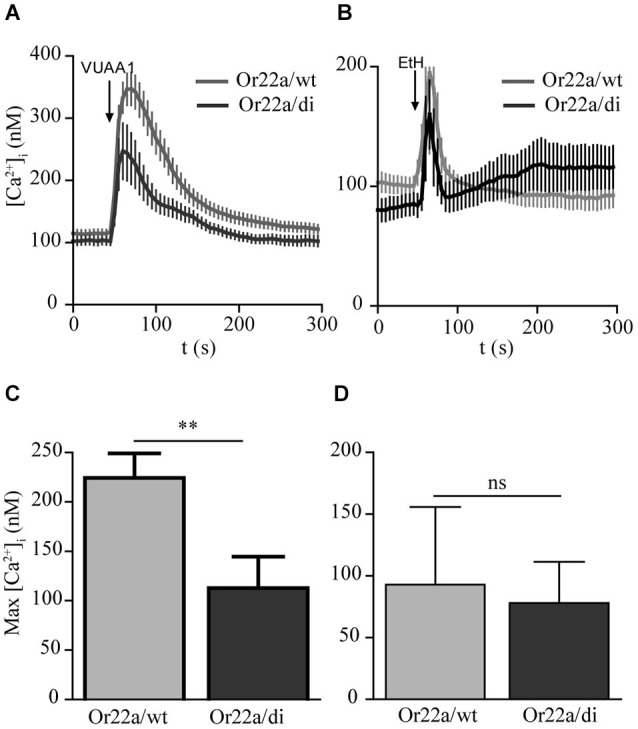
**Calcium responses in cells coexpressing Or22a and Orco wt or Orco di. (A,B)** [Ca^2+^]_i_ upon application of VUAA1 (100 μM) and ethyl hexanoate (EtH, 100 μM) for Or22a coexpressed with Orco wt (VUAA1, *n* = 19 EtH, *n* = 7) and Orco di (VUAA1, *n* = 13 EtH, *n* = 6). **(C,D)** Comparison of maximum [Ca^2+^]_i_ in cells coexpressing Or22a with Orco wt or Orco di as in **(A,B)**. Data represent mean ± SEM; unpaired *t*-test, ** *p* < 0.01, ns, not significant. The curve for Or22a/wt corresponds to a data set shown in Mukunda et al., 2014, Figure 6A.

In heterologous expression systems Orco forms heterodimeric complexes with ligand binding OrX proteins (Neuhaus et al., 2005; Benton et al., 2006). To test whether Orco di interacts with a ligand binding odorant receptor OrX, we next coexpressed Or22a in Orco wt and in Orco di expressing cells and stimulated them with VUAA1 and an Or22a ligand, ethyl hexanoate (Figures 5A,B). With VUAA1stimulation the [Ca^2+^]_i_ signals obtained from cells coexpressing Or22a displayed a slower decay than cells solely expressing Orco, as previously observed (Mukunda et al., 2014). The amplitude of [Ca^2+^]_i_ in Or22a/wt expressing cells was larger than in cells expressing Or22a/di (Figures 5A,C). When stimulated with ethyl hexanoate the amplitudes of Ca^2+^ signals were similar for Orco wt and Orco di expressing cells (Figures 5B,D). The calcium measurements with coexpression of Or22a show that Orco di interacts with OrX proteins to form a functional OR channel. The similar size of odor-induced signals indicates a similar level of functional ORs generated with Orco wt and di, respectively.

## Discussion

Orco is an integral part of insect ORs and is required for the correct insertion of OrX proteins in the dendritic membrane of the receptor neurons (Larsson et al., 2004). In addition to forming heteromers with OrX proteins with an as yet unknown stoichiometry there is also evidence that Orco may build homomeres (Neuhaus et al., 2005; Benton et al., 2006; German et al., 2013). Reports of the purification of insect OR subunits suggest potential dimeric and quaternary structure formation between Orco and Or22a (Carraher et al., 2013). In this study we engineered the minimal oligomeric structure of Orco and asked whether Orco di exhibits the same channel properties like Orco wt.

The calcium imaging experiments with Orco di expressing cells have demonstrated a Ca^2+^ influx in response to non-odor OR agonists such as VUAA1 (Jones et al., 2011; Chen and Luetje, 2012; Figure 2). Heterologously expressed Orco proteins form Ca^2+^ permeable cation channels and show constitutive activity which leads to elevated intracellular [Ca^2+^] resting levels (Sato et al., 2008; Wicher et al., 2008; Jones et al., 2011; Sargsyan et al., 2011). The basal [Ca^2+^]_i_ levels of Orco di expressing cells were also enhanced as compared to non-transfected CHO cells. This indicates also a background activity of Orco di channels (Figure 2F). The agonist stimulation produced Ca^2+^ signals similar to those observed for Orco wt that were dependent on extracellular Ca^2+^. Thus, Orco di shows functional expression and forms a Ca^2+^ permeable cation channel. Whole cell current measurements using patch clamp further confirm that Orco di displays ion channel activity and generates a transient inward current similar to Orco wt when activated by an appropriate ligand (Figure 3).

In a recent study we showed that CaM activity affects the function of Orco channels (Mukunda et al., 2014). Stimulation of Orco wt cells in presence of the CaM inhibitor W7 showed significantly reduced and prolonged [Ca^2+^]_i_ responses. The Orco protein contains a conserved putative CaM binding motif (^336^SAIKYWER^344^) in the second intracellular loop. A point mutation in this putative CaM site (K339N) affects the Ca^2+^ responses elicited by agonist stimulation. As the Orco di protein also contains the putative CaM binding motif it was expected that CaM would regulate this construct. Indeed, the responses obtained with Orco di were similar to those obtained with Orco wt in presence of W7 (Figures 4B,C), suggesting that Orco di is modulated by CaM as Orco wt.

A mutational study of *Bombyx* pheromone receptors suggests that both constituents of olfactory receptors, Orco and OrX proteins contribute to the ion channel pore (Nakagawa et al., 2012). Also, expression of Orco alone leads to functional channels suggesting that they may dimerize which is supported by FRET experiments (German et al., 2013). Coexpression of Orco di and Or22a elicited Ca^2+^ transients in response to Orco agonist VUAA1 and Or22a ligand ethyl hexanoate application as seen in cells expressing Or22a/Orco wt. This suggests an interaction of OrX protein here represented by Or22a (Figure 5). The construction of concatameric GPCR dimers has raised the question whether they would form a functional dimer composed of the two coupled subunits or interconcatameric dimers, i.e., tetramers (Terpager et al., 2009). The first alternative was expected for homomeric constructs such as the β2-adrenergic receptor, but not for a heteromeric couple of β2-adrenergic receptor and neurokinin receptor 1. A similar question arises concerning the composition of Or22a and Orco complex. There might be a tetrameric interaction between two Or22a units and the dimer. Even for Orco di a tetrameric topology cannot be excluded.

In conclusion, our experiments demonstrate that the synthetic Orco di construct is functionally expressed and it forms a functional Ca^2+^-permeable cation channel. Like Orco wt, it can be activated by synthetic agonists like VUAA1 and its derivatives. Furthermore, Orco di seems to be constitutively active leading to enhanced basal [Ca^2+^]_i_ levels in Orco di expressing cells. Finally, our results show that Orco di is modulated by CaM in a similar way as Orco wt and it interacts with OrX proteins such as Or22a. Thus the functional properties of the Orco di construct are very similar to those of Orco wt. This result would be compatible with the assumption that Orco channels build dimeric assemblies. At presence, however, this view requires more support, for example by testing an Orco construct that is prevented to dimerize or by resolving the crystal structure of Orco complexes. An intriguing question is whether Orco di would be able to rescue the Orco function in Orco deficient fly mutants.

## Conflict of interest statement

The authors declare that the research was conducted in the absence of any commercial or financial relationships that could be construed as a potential conflict of interest.

## Footnotes

^1^http://imagej.nih.gov/ij/
